# Similar scapular morphology in patients with dynamic and static posterior shoulder instability

**DOI:** 10.1016/j.jseint.2020.11.003

**Published:** 2021-01-15

**Authors:** Silvan Beeler, Laura Leoty, Bettina Hochreiter, Fabio Carrillo, Tobias Götschi, Tim Fischer, Philipp Fürnstahl, Christian Gerber

**Affiliations:** a Department of Orthopaedic Surgery, University of Zürich, Balgrist University Hospital, Zürich, Switzerland; b Research in Orthopedic Computer Science (ROCS), University of Zürich, Balgrist University Hospital, Zürich, Switzerland; c Unit for Clinical and Applied Research, Balgrist University Hospital, Zürich, Switzerland; d Division of Radiology and Nuclear Medicine, Kantonsspital St. Gallen, St. Gallen, Switzerland

**Keywords:** Posterior shoulder instability, static humeral head subluxation, excentric osteoarthritis, shape of scapula, shape of acromion, lateral acromial roof, acromion morphology

## Abstract

**Background:**

There is evidence that specific variants of scapular morphology are associated with dynamic and static posterior shoulder instability. To this date, observations regarding glenoid and/or acromial variants were analyzed independently, with two-dimensional imaging or without comparison with a healthy control group. Therefore, the purpose of this study was to analyze and describe the three-dimensional (3D) shape of the scapula in healthy and in shoulders with static or dynamic posterior instability using 3D surface models and 3D measurement methods.

**Methods:**

In this study, 30 patients with unidirectional posterior instability and 20 patients with static posterior humeral head subluxation (static posterior instability, Walch B1) were analyzed. Both cohorts were compared with a control group of 40 patients with stable, centered shoulders and without any clinical symptoms. 3D surface models were obtained through segmentation of computed tomography images and 3D measurements were performed for glenoid (version and inclination) and acromion (tilt, coverage, height).

**Results:**

Overall, the scapulae of patients with dynamic and static instability differed only marginally among themselves. Compared with the control group, the glenoid was 2.5° (*P* = .032), respectively, 5.7° (*P* = .001) more retroverted and 2.9° (*P* = .025), respectively, 3.7° (*P* = .014) more downward tilted in dynamic, respectively, static instability. The acromial roof of dynamic instability was significantly higher and on average 6.2° (*P* = .007) less posterior covering with an increased posterior acromial height of +4.8mm (*P* = .001). The acromial roof of static instability was on average 4.8° (*P* = .041) more externally rotated (axial tilt), 7.3° (*P* = .004) flatter (sagittal tilt), 8.3° (*P* = .001) less posterior covered with an increased posterior acromial height of +5.8 mm (0.001).

**Conclusion:**

The scapula of shoulders with dynamic and static posterior instability is characterized by an increased glenoid retroversion and an acromion that is shorter posterolaterally, higher, and more horizontal in the sagittal plane. All these deviations from the normal scapula values were more pronounced in static posterior instability.

## Introduction

Posterior glenohumeral shoulder instability can be divided into “dynamic”, “static”, or a combination of both. In “dynamic” instabilities, the shoulder joint is centered in the resting position and posterior translation of the humeral head occurs by elevating and internally rotating the arm in front of the body and might be more pronounced when tested under load. In “static” instabilities, the shoulder joint is permanently decentered in the resting, supine position, with increased posterior translation by elevation or abduction of the arm.^2^^,^^25^^,^^26^ Static posterior humeral head subluxation is considered to be arthrogenic and a probable cause of primary eccentric osteoarthritis associated with B1, B2, or B3 glenoid deformation according to Walch.^6^^,^^25^^,^^26^

So far, recurrent posterior shoulder instability and static posterior humeral head subluxation are understood as two distinct pathologic conditions, which become symptomatic in different periods of life. Although the pathogenesis remains multifactorial and—hitherto—poorly understood, there is increasing evidence that the scapular shape could play a relevant role in both conditions^2^^,^^16^^,^^17^^,^^21^^,^^26^^,^^28^: Increased glenoid retroversion has been discussed extensively, but on its own is unable to comprehensively explain the observations.^2^^,^^21^^,^^26^^,^^28^ More recently a flat acromial roof with less posterior bony support has been observed and described as an anatomical variant which is strongly associated with posterior instability.^2^^,^^16^^,^^20^ Up to now, these observations were analyzed either independently of each other, two-dimensionally^16^^,^^17^ or without a healthy control group.^2^

The goal of this study was therefore to analyze the three-dimensional (3D) scapular morphology of normal shoulders and shoulders of patients with dynamic and static posterior shoulder instability. We hypothesized, that in addition to an increased retroversion of the glenoid, posterior instability is also associated with a higher, and flatter acromion than the normal values on a typical normal shoulder. This may contain important information for better understanding of the pathologic role of the scapula, which could be either a dependent or an independent common denominator in the development of dynamic and static posterior shoulder instability.

## Materials and methods

### Materials

#### Study design

This is a retrospective case-control study involving 1) patients with dynamic posterior shoulder instability (group 1), 2) patients with static posterior shoulder instability (group 2), and 3) patients without any clinical symptomatology or morphological pathology of the shoulder (group 3). Approval was obtained from the responsible ethical committee (Kantonale Ethikkommission Kanton Zürich, Schweiz BASEC-Nr 2020-00389).

#### Patient population

**Figure 1:**
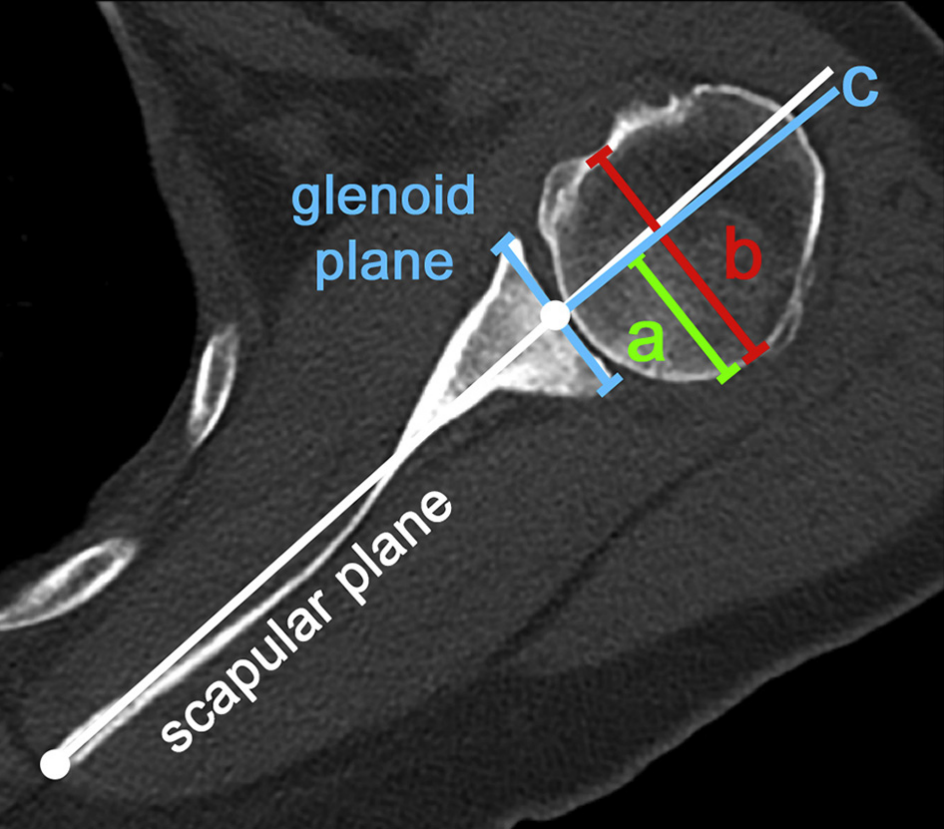
Glenohumeral subluxation index: Glenohumeral subluxation index in % = a/b. Measurement performed on midglenoid plane. a = relative part of the humeral head posterior to c, b = humeral head diameter, c = line bisecting the glenoid.
• Group 3 (control group): For the healthy control group, we selected 40 patients (20 women and 20 men) between age 45 to 65 years without any shoulder pathology. The CT scans were performed in the course of a polytrauma treatment. CT scans with visible bony defects of the scapula/humerus, osteoarthritis, rotator cuff tears, glenoid dysplasia (Walch type C glenoid and glenoid dysplasia according to Weishaupt)^26^^,^^28^ and a history of any shoulder pathology in the past were excluded.

• Group 1 (dynamic posterior instability): We retrospectively reviewed all patients who had been treated operatively for unidirectional, recurrent posterior shoulder instability between January 2007 to January 2020 in our institution. Inclusion criteria were a positive clinical examination for posterior shoulder instability (positive jerk test^12^ and/or posterior apprehension test^30^), specific intraoperative findings (i.g., posterior labral lesions, wide posterior capsule), and available CT scanning of the scapula. Excluded were patients with unsatisfactory CT scan (missing medial scapular border), previous surgical interventions of the affected shoulder, any bony glenoid rim or humeral head defects (i.g., reversed bony Bankart lesion, glenoid fractures, Hill-Sachs lesions, malunions after fracture, bony exostosis), multidirectional instability,^9^ glenoid dysplasia (Walch type C glenoid and glenoid dysplasia according to Weishaupt),^26^^,^^28^ connective tissue disorders, rotator cuff tears and immature skeletal age (open physes in CT scan). Of 168 identified shoulders with unidirectional posterior shoulder instability, only 30 shoulders (from 30 different patients) fulfilled the group's inclusion criteria. Exclusion reasons: 57 shoulders with missing or incomplete CT scan, 25 previous surgery, 9 bony glenoid rim or humeral head defects, 47 other reasons.
• Group 2 (static posterior instability): We retrospectively screened our database for patients with eccentric osteoarthritis of the posterior glenoid and a glenohumeral subluxation index of >55% (static posterior humeral head subluxation measured in midglenoid plane^1^^,^^2^), representing a Walch type B1 glenoid^25^^,^^26^ (Figure 1). Excluded were patients with unsatisfactory CT scans, previous surgical interventions on the affected shoulder, shoulder instability (history of previous shoulder dislocation, anterior/posterior apprehension), advanced osteoarthritis (Walch type B2 or B3 glenoid),^25^ glenoid dysplasia (Walch type C glenoid and glenoid dysplasia according to Weishaupt),^26^^,^^28^ or secondary osteoarthritis (rotator cuff tear, rheumatoid arthritis, avascular necrosis, instability, infection, or fracture). Unfortunately, we could only identify 9 shoulders of 9 patients in our clinic. But we could include a further 11 shoulders of 11 patients through a collaboration with Dr. Gilles Walch Lyon (France) and Drs. JP Iannotti and E. Ricchetti (Cleveland, clinic (USA)). Group 2 consisted thus of 20 patients with 20 shoulders.

All patient characteristics (including age and gender) can be found in Table I.

**Table I:** Patient characteristics

Patient characteristics of group 1, 2, and 3	Group 1: Dynamic (N = 30)	Group 2: Static (N = 20)	Group 3: Control (N = 40)
Age (y)	22.5 (SD ± 4.94)	59.8 (SD ± 12.26)	51.8 (SD ± 5.61)
Gender (F/M)	7/23 (24%/77%)	13/7 (65%/35%)	20/20 (50%/50%)
Side (R/L)	13/17 (43%/57%)	8/12 (40%/60%)	20/20 (50%/50%)

### Methods

The individual's CT scan was imported into MIMICS software (version 22.0, Materialise, Leuven, Belgium) to perform semiautomatic thresholding segmentation and 3D model generation. Further calculations were performed in the in-house developed planning software CASPA (Computed Assisted Surgery Planning Application, version 5.0) in combination with MATLAB (2019b, The MathWorks, Inc., Natick, MA, USA). The 3D measurements were performed by three of the authors (L.L., B.S., B.H.).

#### Scapular coordinate system

**Figure 2:**
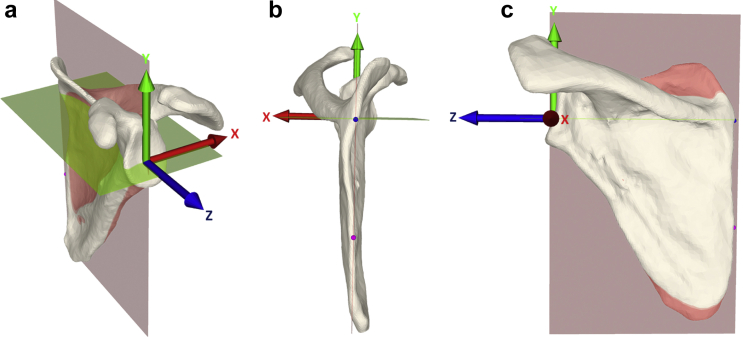
Landmarks, glenoid center, supraspinous fossa line: (**a**) Landmarks: 10 anatomical landmarks were visually placed on the scapula: 6 (*red points*) were placed on the inferior part of the glenoid rim, 1 (*cyan point*) at the beginning of the supraspinous fossa, 1 (*yellow point*) at its end, 1 (*blue point*) at the spinoglenoidal intersection and 1 (*pink point*) at the inferior-medial scapular margin. Because some of the CT scans did not include the inferior tip of the scapula, the inferior-medial scapular point was set inferior of the spinoglenoidal intersection point, at a distance of 2 times of the glenoid diameter of the best circle fitting of the glenoid. (**b**) Glenoid center: Best circle fit of the inferior glenoid landmarks (6 *red points*). The center of the circle was projected on the glenoid surface to define the glenoid center, which constitutes the center of the scapular coordinate system. (**c**) Supraspinous fossa line: A local coordinate system was generated with the y-axis being the line between the 2 landmarks of the fossa, the z-axis being the medial borderline between the 2 landmarks of the medial border projected on the plane normal to the y-axis. The x-axis is mutually perpendicular to the y and z-axis. The plane was translated along the y-axis and for each position; the point having the minimum z coordinate was extracted. The supraspinous fossa is the line fitting on all the selected points.

**Figure 3:**
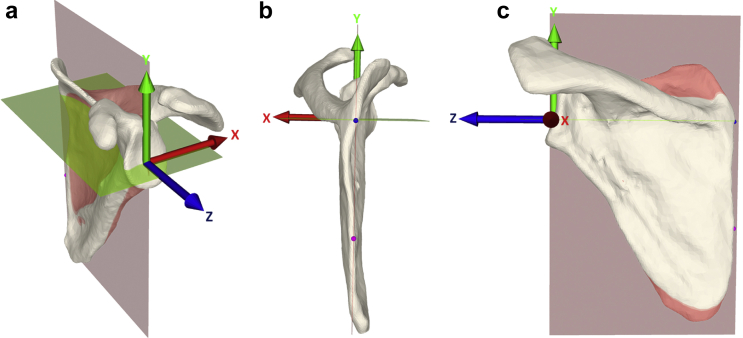
Scapular coordinate system (**a-c**): the center of the best-fit circle of the inferior glenoid (see Figure 1*b*), the spinal medial point, and the point placed at a standardized distance to the spinal medial point are defining the scapular plane (x-axis shown in red and scapular plane shown in *pink*); the supraspinous fossa direction (see Figure 1*c*) projected on the scapular plane defines the z-axis (shown in *blue*), and the y-axis (shown in *green*) is mutually perpendicular to the x- and z-axis. All 3D-reconstructed scapulae were aligned to this scapula-based coordinate system.

First of all, a scapular coordinate system was created, based on 10 anatomical landmarks, which were placed visually on the scapula (Figure 2*a*). The origin of the coordinate system was set at the glenoid center. The latter was calculated as the center of the best fit circle obtained from the 6 inferior landmarks placed on the glenoid rim (red spheres, Figure 2*b*), and projected onto the glenoid surface. The scapular plane (pink plane in Figure 3) was defined as the plane going through the two landmarks of the medial scapular border (Figure 2*a*; blue and pink points) and the glenoid center (Figure 2*b*). The posterior-anterior x-axis was perpendicular to the scapular plane. The mediolateral z-axis (blue arrow in Figure 3) was defined as the line (green line in Figure 2*c*) fitted automatically through the points of the supraspinous fossa line (black points in Figure 2*c*), projected on the scapular plane. These points were defined as the surface points of the supraspinous fossa between the two landmarks of the medial points (cyan and yellow points in Figure 2*a*). The infrasuperior y-axis was mutually perpendicular to the x- and z-axis. Figure 3 shows the calculated coordinate system, defined for a left scapula. Consequently, the right scapulae were all mirrored. Hereby, the following planes could be defined; xy-plane = medial plane shown in green in Figure 3, xz-plane = axial plane, yz-plane = scapular or sagittal plane.

#### Measurement parameters

**Figure 4:**
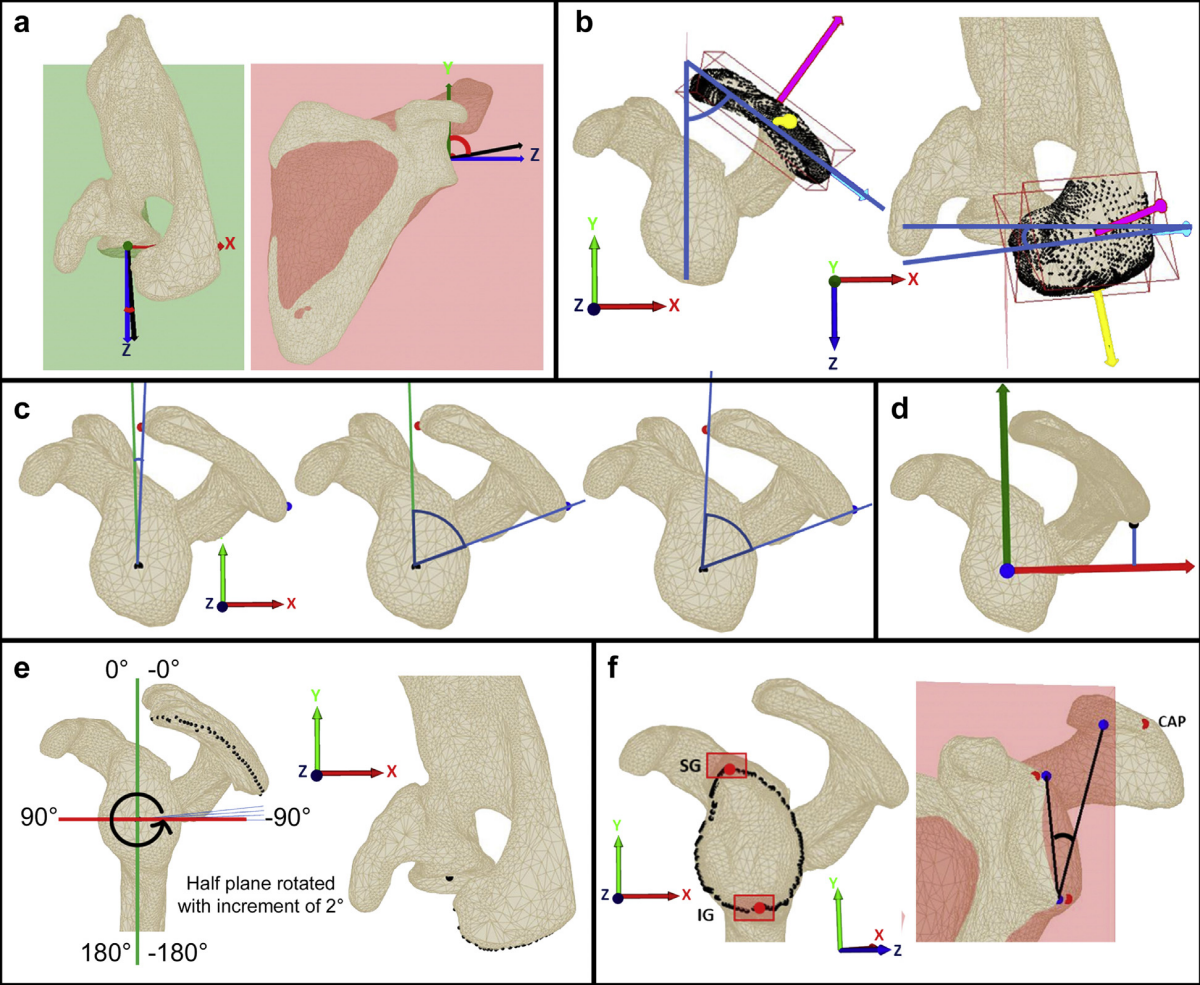
Measurement parameters. (**a**) Glenoid version and inclination: Version = angle between the glenoid plane normal and the z-axis projected on the xz-plane (*left*). Inclination = angle between the glenoid plane normal and the y-axis projected on the yz-plane i.e the scapular plane (*right*). (**b**) Sagittal and axial tilt: Sagittal tilt = angle between the y-axis of the scapular coordinate system and the length axis of the oriented bounding box of the acromion (shown in *light blue*), projected on the xy-plane of the scapular coordinate system (*left*). Axial tilt = angle between the (posterior-anterior) x-axis of the scapular coordinate system and the length axis of the oriented bounding box of the acromion (shown in *light blue*), projected on the xz-plane of the scapular coordinate system (*right*). (**c**) Glenoid coverage: anterior coverage (*left*), posterior coverage (*middle*), and overall coverage (*right*). (**d**) Lateral coverage, acromial height: Acromion lateral points. A plane orthogonal to the scapular plane (−90°) was created and rotated in the counterclockwise direction with an increment of 2°. For each position, the intersection points with the scapula were extracted (if there was any) and the one having the maximum z-coordinate was taken as the lateral point for this angle. (**e**) Posterior acromial height: The posterior acromial height was defined as the y-coordinate of the acromion point having the minimum y-coordinate. (**f**) Critical shoulder angle (CSA): The 3D CSA was acquired in a semiautomated method calculating the most lateral points of the acromion (CAP), the inferior glenoid point (IG), and the superior glenoid (SG) by selecting the contour points and extracting the most lateral points among the inferior and superior pre-selection of points (*red points*, left figure). The angle between CAP, IG, and SG was transformed into a 2D angle by projecting the points on the yz-plane (*blue points*, right figure).
2) Glenoid inclination was defined as per the β-angle of Maurer^15^ in relation to the supraspinous fossa and measured as the angle between the normal of the glenoid plane projected on the yz-plane and the y-axis of the coordinate system. If the projected glenoid plane normal is oriented toward cranial, the inclination is less than 90°, if it is oriented toward caudal, the inclination is more than 90° (Figure 4*a*, right).
3) Acromion sagittal and axial tilt: The acromion points were automatically selected as the points that are lateral to the glenoid plane. A 3D-oriented bounding box was generated with these points using the method described by Letta C.^14^ The sagittal tilt^1^^,^^2^ was calculated as the angle between the y-axis and the length axis of the bounding box (shown in light blue in Figure 4*b*) of the acromion projected on the xy-plane of the scapular coordinate system. The smaller the sagittal tilt, the steeper the acromion roof is. The axial tilt^1^^,^^2^ was calculated as the angle between the posterior-anterior x-axis and the length axis of the bounding box of the acromion projected on the xz-plane. The smaller the axial tilt is, the more internally rotated the acromion is (Figure 4*b*). The coordinate system shown represents the 3 main principal components of the bounding box, that is, the 3 main directions on which the acromion points are distributed: length (shown in light blue), width (shown in yellow), and height (shown in pink). For the sagittal and axial tilst, only the length axis of the bounding box was used, as it is the axis which describes the largest point distribution.
4) Anterior glenoid coverage: the anterior glenoid coverage^1^^,^^2^ was defined as the anterior extension of the acromion roof about the scapular plane. The point of the acromion having the minimum x-coordinate (anterior point) was taken and the angle between the y-axis of the coordinate system and the line connecting the anterior point with the glenoid center projected on the xy-plane was defined as the anterior glenoid coverage. If the acromion roof extended anterior to the scapular plane, the value was positive, if not, it was negative (Figure 4*c*, left).
5) Posterior glenoid coverage: the posterior glenoid coverage^1^^,^^2^ was defined as the posterior extension of the acromion roof about the scapular plane. The point of the acromion having the maximum x-coordinate (posterior point) was taken and the angle between the y-axis of the coordinate system and the line connecting the posterior point and the glenoid center projected on the xy-plane was defined as the posterior glenoid coverage (Figure 4*c*, middle).
6) Overall glenoid coverage: The overall glenoid coverage^1^^,^^2^ was defined as an angle between a first line defined by the anterior point of the acromion and the glenoid center and a second line defined by the posterior point and the glenoid center, projected on the xy-plane (Figure 4*c*, right).
7) Lateral glenoid coverage and lateral acromial roof height: The positions of the acromial roof were compared by extracting the lateral points of the acromion from the scapula. The most lateral points of the acromion were identified by creating a plane that was orthogonal to the scapular plane and centered at the glenoid center. The plane was rotated around the z-axis of the coordinate system clockwise with increments of 2°. For each angle position, the intersection points between the plane and the acromion were calculated and the one having the maximum z-coordinate (i.e, the most lateral one) was taken as the most lateral point for this plane orientation. The lateral glenoid coverage was calculated as the distance between the glenoid center point, which was projected to the xy-plane, to each intersection point. And the lateral acromial height was calculated as the distance between the glenoid center point to each intersection point, which were projected onto the xy-plane. To normalize the values, the humeral head radius was subtracted.

1) Glenoid version was defined as the angle between the normal vector of the glenoid plane projected on the xz-plane and the z-axis of the coordinate system. If the projected glenoid plane normal is oriented toward posterior, the version is negative (Figure 4*a*, left).

Finally, all points of the z-coordinate direction (lateral glenoid coverage) and xy-coordinate direction (lateral acromial roof height) were visualized as a mean curve for each group (Figure 4*e*).

8) Posterior acromial height: The posterior acromial height^16^ was defined as the y-coordinate of the acromion point having the minimum y-coordinate (Figure 4*e*).
9) Critical Shoulder Angle (CSA): The CSA (critical shoulder angle) was measured according to Moor^18^ as the angle between the most lateral point of the acromion and the inferior glenoid margin and superior glenoid margin. The most lateral point of the acromion called critical point acromion was obtained in an automatic fashion as the point of the acromion having the maximum z-coordinate. The glenoid surface points were selected in a semi-automatic way and enabled to extract the glenoid contour points. The inferior and superior glenoid margins were obtained by preselecting the inferior and superior points of the glenoid contour points and extracting the ones having the maximum z-coordinates of the 2 preselections (Figure 4*f*).
10) Humeral head diameter: The humeral head diameter was measured as the best circle-fit on the axial view of the CT.^29^^,^^32^

### Statistics

To quantify the agreement between the two raters, intraclass correlation coefficients (ICC) were computed for all performed 3D measurements. ICCs were based on a 2-way random effects model describing the absolute agreement of single measurements. ICC estimates and associated 95% confidence intervals are reported. To investigate differences in the radiological parameters between the outcome groups, logistic regression models were used. The single-term models estimate the probability of each case being in either instability group, given the respective measurement. Both instability groups were compared with the control group in separate analyses. Risk analysis was based on the average of both readers per measurement. Finally, 3D trajectories were compared at each sampled position with an ANOVA and post hoc pairwise T-test comparisons. The analysis was conducted with MATLAB (2019b, The MathWorks, Inc., Natick, MA, USA) and SPSS (IBM SPSS Statistics for Windows, Version 25.0. Armonk, NY, USA). *P* values below .05 were considered statistically significant.

## Results

At the time of examination, patients with dynamic posterior shoulder instability were on average 37 years younger than patients with static posterior shoulder instability (*P* < .001). Men were predominant in dynamic and women in static posterior shoulder instability (*P* < .001). There were no significant side differences (*P* = ns). All patient characteristics can be found in Table I.

### Dynamic instability vs control group

**Figure 5:**
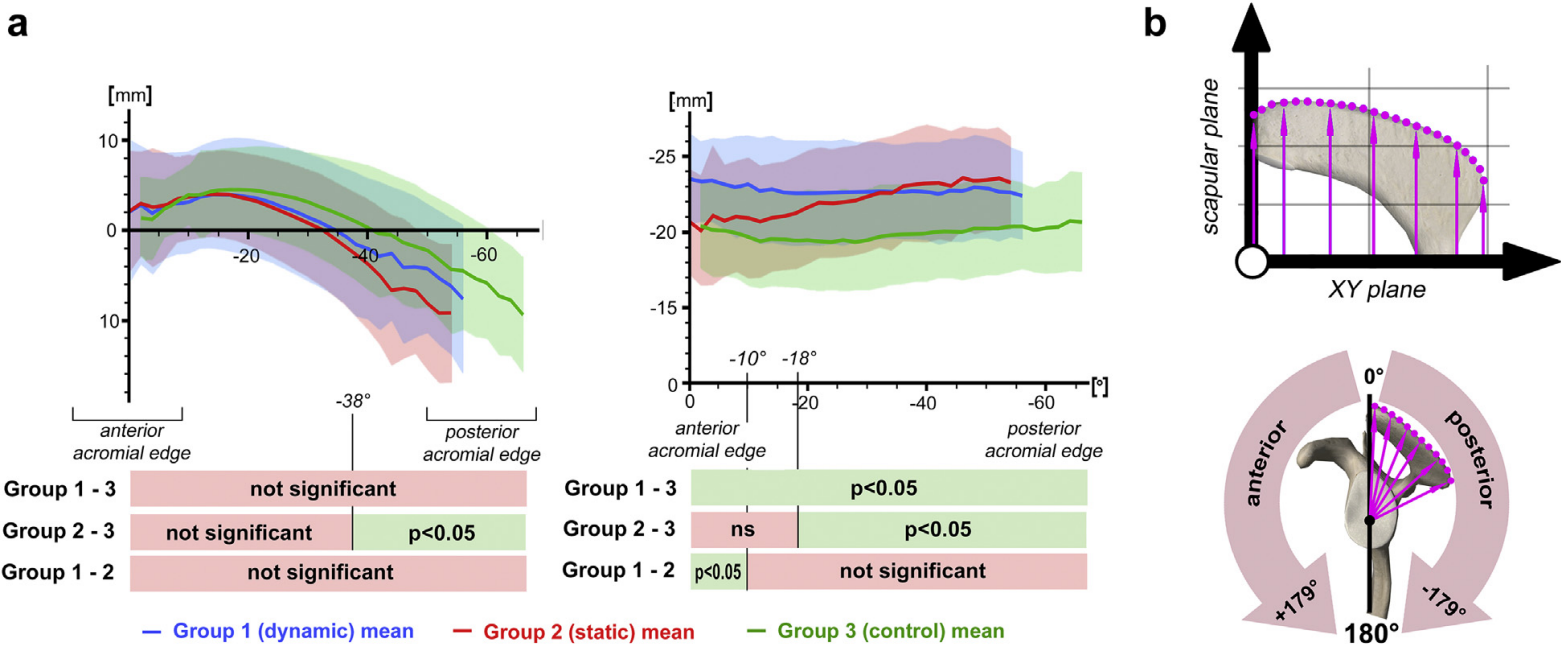
Lateral acromial coverage and lateral acromial height. (**a**) Table with visualized lateral acromial coverage and lateral acromial height. The mean curve per group is plotted from the mean angle of the anterior point of the acromial edge to the mean angle of the most posterior point of the acromial edge. *Blue curve* (mean values of group 1; dynamic instability) with *blue bar* (standard deviation). *Red curve* (mean values of group 2; static instability) with *red bar* (standard deviation). *Green curve* (mean values of group 3; control) with *green bar* (standard deviation). (**b**) Explanation of radial measurement of all lateral acromial points, whereby positive (+) values (from 0° to +180°) are anterior to the scapular plane and negative (−) values (from 0° to −180°) posterior. Example points (*pink points*) with corresponding measurement distances (*pink arrows*).

The glenoid of patients with dynamic instability had on average an increased retroversion of 2.5° (*P* = .032) and increased downward inclination of 2.9° (*P* = .025). The acromion roof was significantly higher (details see Figure 5) and on average 6.2° (*P* = .007) less posterior covering with an increased posterior acromial height of +4.8 mm (*P* = .001). The acromial orientation (axial tilt and sagittal tilt) was not statistically significant. The CSA was reduced by 3.7° (*P* = .001). All values can be found in Table II and Figure 5.

**Table II:** Outcome values

Outcome	Group 1: Dynamic (D) (N = 30)	Group 2: Static (S) (N = 40)	Group 3: Control (C) (N = 40)	D vs C	S vs C	D vs S
Version	−7.4° *(SD ± 4.3°)*	−10.6° *(SD ± 5.4°)*	−4.9° *(SD 4.9°)*	**0.032**	**0.001**	**0.037**
Inclination	82.6° *(SD ± 6.0°)*	83.3° *(SD ± 5.7°)*	79.6° *(SD ± 4.3°)*	**0.025**	**0.014**	0.604
Axial tilt	28.8° *(SD ± 10.4°)*	32.2° *(SD ± 9.5)*	27.4° *(SD ± 7.3)*	0.493	**0.041**	0.247
Sagittal tilt	59.3° *(SD ± 9.7)*	63.0° *(SD ± 8.5)*	55.7° *(SD ± 7.6)*	0.091	**0.004**	0.173
Anterior coverage	0.6° *(SD ± 8.6)*	1.8° *(SD ± 8.2)*	−0.3° *(SD ± 7.7°)*	0.647	0.321	0.602
Posterior coverage	56.7° *(SD ± 9.5)*	54.6° (SD *±* 6.7)	62.9° *(SD ± 7.5)*	**0.007**	**0.001**	0.376
Overall coverage	57.3° *(SD 6.1)*	56.4° *(SD ± 6.5)*	62.6° *(SD ± 6.1)*	**0.002**	**0.003**	0.609
Posterior acromial height	20.3 mm *(SD ± 5.6)*	21.3 mm *(SD ± 4.2)*	15.5 mm *(SD ± 4.9)*	**0.001**	**0.001**	0.523
Critical shoulder angle (CSA)	28.0 *(SD ± 3.8)*	25.2 *(SD ± 4.1)*	31.7 *(SD ± 3.6)*	**0.001**	**<0.001**	**0.028**
Humeral head diameter	44.4 (SD *±* 2.7)	43.1 (SD *±* 3.8)	44.6 (SD *±* 3.4)	0.693	0.133	0.177

Significant values are marked in bold.

### Static instability vs control group

The glenoid of patients with static instability had on average an increased retroversion of 5.7° (*P* = .001) and an increased inclination of 3.7° (*P* = .014). The acromion was on average 4.8° (*P* = .041) more “externally rotated” (axial tilt), 7.3° (*P* = .004) flatter (sagittal tilt), 8.3° (*P* = .001) less posteriorly covered, and posteriorly 5.8 mm (*P* = .001) higher (posterior acromial height). Furthermore, the acromial roof was less covering laterally (lateral acromial coverage ≤−32°, *P* < .05) and higher (lateral acromial height ≤−38°, *P* < .05) in the posterior parts of the acromion compared to the control group. The CSA was on average 6.5° (*P* < .001) smaller. All values can be found in Table II and Figure 5.

### Dynamic instability vs static instability

All values were more pronounced in the static than the dynamic posterior shoulder instability groups. But statistically significant differences could only be found for glenoid retroversion (*P* = .037), CSA (*P* = .028), and for the anterior 10° of the lateral acromial height. All values can be found in Table II.

### Gender influence

There were no gender differences in all three groups for all measurement parameters. The Pearson correlation index was ρ = 0.219 for our control group, ρ = 0.79 for dynamic instability, and ρ = −0.299 for static instability.

### Inter-reader reliability

All 3D measurements had an excellent inter-reader reliability (ICC = 0.982 to 0.999).

## Discussion

**Figure 6:**
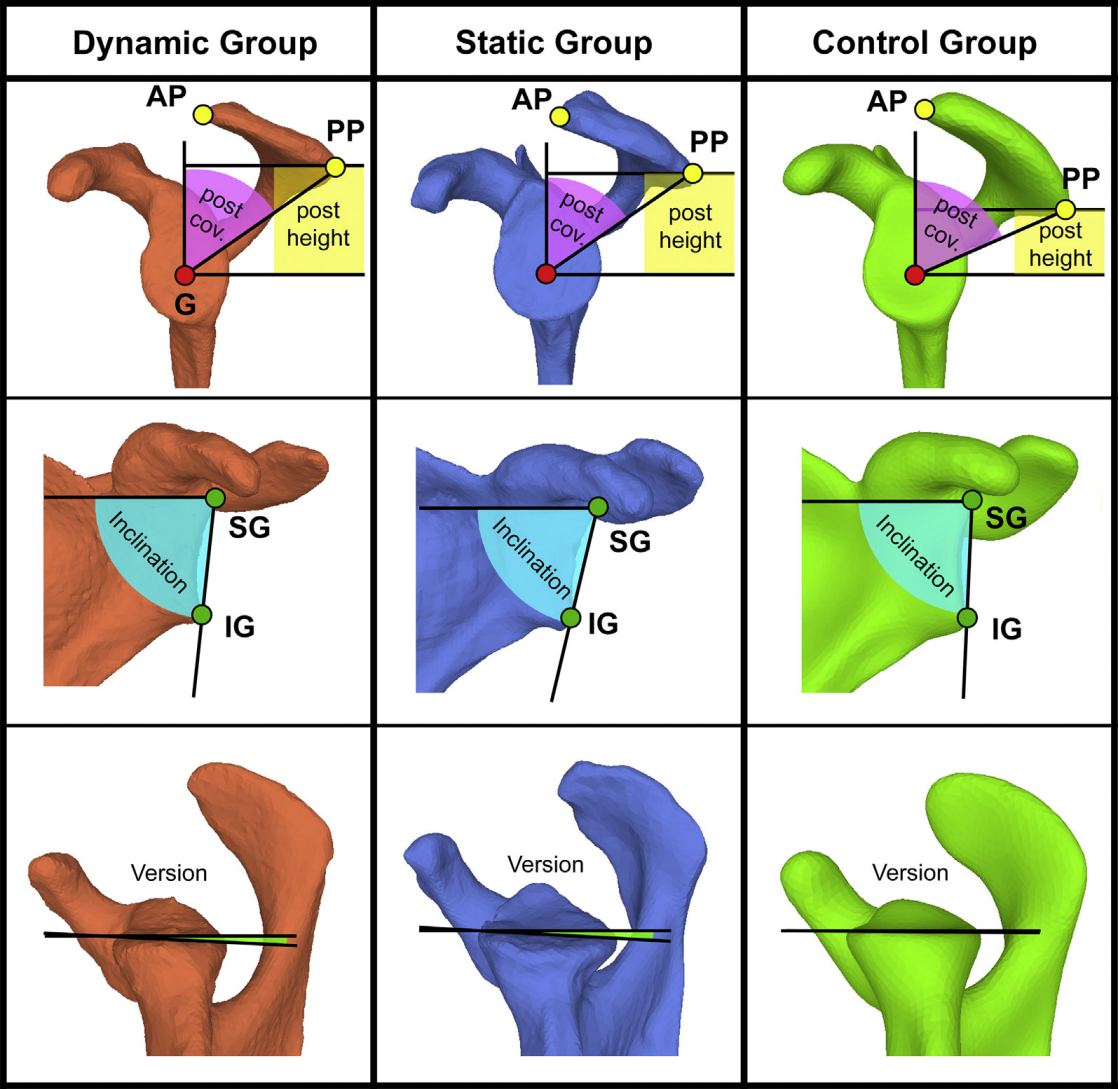
Differences in scapular morphology: Typical examples of the scapular shape of dynamic posterior instability, static posterior instability, and our control group. Increased posterior acromial height (=*yellow*; post height), decreased posterior acromial coverage (=*pink*; post coverage), increased downward inclination (=*cyan*; inclination), and increased glenoid retroversion (=*green*; version) in static and dynamic posterior instability compared to the control group. *AP*, anterior acromial point; *PP*, posterior acromial point; *SG*, superior glenoid point; *IG*, inferior glenoid point.

This is the first study that compares the scapular morphology of normal, static, and dynamic glenohumeral instabilities in 3D. Interestingly, the scapular morphology of dynamic posterior shoulder instability and static posterior humeral head subluxations was similar (Figure 6). In both conditions, the glenoid was more retroverted and downward tilted, and the acromial roof was flatter (sagittal tilt) with decreased posterolateral coverage, compared with our healthy control group. These findings are in consent with previous studies, which could find an increased glenoid retroversion,^2^^,^^7^^,^^8^^,^^11^^,^^13^^,^^21^^,^^25^^,^^28^ and flat acromial roof with increased posterior acromial height^2^^,^^16^^,^^17^ for static and dynamic posterior instability. Considering the mean values, all these differences were more pronounced in static instability, but only significantly more pronounced for glenoid retroversion (Δ 3.2°; *P* = .037) and CSA (Δ 2.8°; *P* = .028). Furthermore, the acromial roof was higher in both instability groups. While this applied for the entire acromial roof in dynamic instability, only the posterior part of the acromion was higher in static instability.

So far, dynamic posterior shoulder instability and static posterior humeral head subluxation are both considered as two distinct pathologic conditions. Recurrent posterior shoulder instability begins at young age (in our study first symptoms on average with 19 years) without or after a minor traumatic event. Patients report often pain or vague posterior-based shoulder discomfort with or without associated catching or clicking. Discomfort would be increased by placing the arm in the provocative position in front of the body.^23^ On the other side, patients with eccentric posterior glenoid wear due to static posterior humeral head subluxation are much older at the beginning of the first symptoms (in our study first symptoms with age 50), they report of progressive functional impairment rather than shoulder hyperlaxity,^25^ and previous events of shoulder dislocation or instability are usually denied.^25^ However, despite all these facts, an association between both conditions may still be possible. First, the natural history of dynamic posterior shoulder instability is still unknown, but the development of eccentric osteoarthritis after posterior shoulder stabilization is frequently seen, of course unknown whether as a cause of surgery or consequence of recurrency.^3^ Second, pain and a feeling of instability/discomfort are often reported not only in dynamic, but also in static posterior instability.^25^ And because posterior shoulder instability is very difficult to diagnose, pain could be misinterpreted. Third, static humeral head subluxation is not only a “static” condition. Rather posterior translation of the humeral head increases by shoulder flexion and abduction,^2^^,^^24^ which equals a dynamic posterior shoulder subluxation. Fourth, proof of concept of posterior humeral head subluxation as the first sign of eccentric osteoarthritis is still missing. Although a progression over time could be recently shown for eccentric osteoarthritis with type Walch B1 transitioning to type B2,^27^ there is no proof that static posterior humeral head subluxation without degenerative changes (Walch B0)^6^ is the first appearance of this disease. As it must be assumed that Walch B0 glenoid is asymptomatic, this question could probably never be entirely answered. It is made even more difficult because noticed posterior subluxation of the humeral head can not be seen automatically as the beginning of eccentric osteoarthritis.^33^ And fifth, as shown in this study, the scapular shape seems to be very similar. Glenoid retroversion is already known as an important factor in humeral head decentration.^10^ And a flat acromial roof with higher posterior roof and less postero-lateral glenoid coverage could have less posterior resistance for humeral head centralization^2^ with a possible unfavorable force vector of the deltoid muscle. Both anatomical factors together could be an unfavorable scapular condition which could become symptomatic either early as dynamic posterior shoulder instability in the active younger generation with increased risk for minor or major traumatic events of damaging the posterior capsule and/or labrum. Or even later as static posterior humeral head subluxation with already visible posterior glenoid wear. Because particularly young men are more risk-averse and physically active, this could explain why young men had more dynamic and older women more static posterior shoulder instability in our cohort.

But if scapular morphology would be really the primary factor in the etiology of posterior instability, first symptoms should be present the earlier the more pronounced these factors would be. However, patients with static posterior instability had more pronounced changes but were on average 37 years older than patients with dynamic instability. Either that means dynamic and static shoulder instability are two distinct pathologic conditions with similar morphological scapular shapes or the morphology of posterior instability is still increasing with age, as a pathologic adaptative osseous process.

In the following, we discuss some of the limitations of this work. The medical history (including pain, instability, and first episodes) was based on prior medical documentation. And hyperlaxity, as a potential risk factor of instability, could not be retrospectively analyzed or excluded. Unfortunately, our groups were inhomogenous with regard to sample size, age, and gender. Despite intensive searching, we could only identify 30 patients with unidirectional dynamic shoulder instability and 20 patients with isolated static posterior humeral head subluxation. Several cases had to be excluded because of missing/inadequate CT scans, previous surgical interventions, multidirectional instability, or other reasons. Because age and gender were predetermined and substantially different in both instability groups, it was not possible to choose a gender- and age-matched control group. We finally decided on a control group with gender equality and an age between 45 and 65 years old. We herewith assured the unintentional inclusion of shoulders that still become dynamically unstable (age <45), and shoulders with already age-related degenerative changes of the scapula (age >65). Although this has to be noticed as a limitation of this study, we could not find any statistical differences between age and gender among any of the three groups. Finally, to minimize influences by different scapular sizes, we subtracted the humeral head diameter of the measured lateral acromial height and lateral acromial coverage.

In summary, this study does not maintain that dynamic shoulder instability and static humeral head subluxation are the same pathologic condition. However, it could be shown that the scapular shape is similar between these two pathologic conditions and distinctly different from normal shoulders. Therefore, other unknown factors—such as probably ligament laxity,^5^^,^^8^^,^^31^ muscle imbalance,^4^^,^^7^^,^^19^^,^^24^^,^^27^ or even humeral anatomy^22^—may be responsible, for the development of either condition.

## Conclusion

The scapular morphology of shoulders with dynamic posterior shoulder instability and with static posterior humeral head subluxation is very similar, and clearly different from normal shoulders. Both conditions are associated with increased glenoid retroversion and an acromion which is higher and less covering posterolaterally. All these variants were more pronounced in static instability.

## Acknowledgments

Special acknowledgments to Dr. Gilles Walch Lyon (France) and Drs. JP Iannotti and E. Ricchetti (Cleveland Clinic (USA)) for their contributions in patient selection.

## Disclaimer

The authors, their immediate families, and any research foundations with which they are affiliated have not received any financial payments or other benefits from any commercial entity related to the subject of this article.
